# Supplementary material for: Hot spot prediction in protein-protein interactions by an ensemble system
Source: BMC Syst Biol. 2018 Dec 31;12(Suppl 9):132. doi: 10.1186/s12918-018-0665-8 (PMC6311905; doi:10.1186/s12918-018-0665-8)
Supplement: Supplementary file 2 — All relevant MATLAB code. A simple Matlab implement of our predictor. (ZIP 19,742 kb) [file 12918_2018_665_MOESM2_ESM.zip › Additional file 2/Read ASA/ASA website/SKEMPI/1VFBA.htm]

 NetSurfP - Protein Surface Accessibility and Secondary Structure Predictions

  

|  |  |  |
| --- | --- | --- |
|  |  | NetSurfP - Protein Surface Accessibility and Secondary Structure Predictions  Technical University of Denmark |

  


---

```
# For publication of results, please cite:
# A generic method for assignment of reliability scores applied to solvent accessibility predictions.
# Bent Petersen, Thomas Nordahl Petersen, Pernille Andersen, Morten Nielsen and Claus Lundegaard
# BMC Structural Biology 2009, 9:51 doi:10.1186/1472-6807-9-51
#
# Column 1: Class assignment - B for buried or E for Exposed - Threshold: 25% exposure, but not based on RSA
# Column 2: Amino acid
# Column 3: Sequence name
# Column 4: Amino acid number
# Column 5: Relative Surface Accessibility - RSA
# Column 6: Absolute Surface Accessibility
# Column 7: Z-fit score for RSA prediction
# Column 8: Probability for Alpha-Helix
# Column 9: Probability for Beta-strand
# Column 10: Probability for Coil
E D  1VFBA                  1    0.802 115.583   0.507   0.003   0.003   0.994
B I  1VFBA                  2    0.280  51.837  -0.793   0.004   0.514   0.481
E V  1VFBA                  3    0.450  69.196   0.951   0.002   0.816   0.182
B L  1VFBA                  4    0.153  27.923   0.564   0.002   0.816   0.182
E T  1VFBA                  5    0.422  58.587   0.382   0.003   0.718   0.279
B Q  1VFBA                  6    0.147  26.343   0.348   0.004   0.514   0.481
E S  1VFBA                  7    0.391  45.778   0.147   0.004   0.138   0.858
E P  1VFBA                  8    0.449  63.713  -0.848   0.018   0.019   0.964
E A  1VFBA                  9    0.521  57.436  -1.227   0.053   0.043   0.903
E S  1VFBA                 10    0.361  42.333  -1.140   0.066   0.296   0.638
B L  1VFBA                 11    0.222  40.612  -0.126   0.023   0.655   0.322
E S  1VFBA                 12    0.414  48.521  -0.572   0.021   0.756   0.223
B A  1VFBA                 13    0.238  26.195  -0.084   0.023   0.655   0.322
E S  1VFBA                 14    0.417  48.849   0.404   0.022   0.359   0.619
E V  1VFBA                 15    0.507  77.987   0.085   0.004   0.085   0.910
E G  1VFBA                 16    0.647  50.911  -1.353   0.005   0.045   0.951
E E  1VFBA                 17    0.382  66.788   0.349   0.004   0.138   0.858
E T  1VFBA                 18    0.544  75.494   1.119   0.003   0.718   0.279
B V  1VFBA                 19    0.075  11.558  -0.116   0.001   0.959   0.040
E T  1VFBA                 20    0.415  57.505   1.278   0.001   0.959   0.040
B I  1VFBA                 21    0.051   9.435   0.340   0.001   0.959   0.040
E T  1VFBA                 22    0.364  50.431   0.600   0.001   0.900   0.099
B C  1VFBA                 23    0.027   3.763   0.301   0.002   0.816   0.182
E R  1VFBA                 24    0.432  99.020   1.135   0.002   0.816   0.182
B A  1VFBA                 25    0.267  29.467  -1.341   0.004   0.616   0.381
E S  1VFBA                 26    0.358  41.934  -1.526   0.004   0.420   0.576
E G  1VFBA                 27    0.501  39.444  -2.468   0.004   0.085   0.910
E N  1VFBA                 28    0.448  65.587  -2.423   0.018   0.047   0.935
E I  1VFBA                 29    0.418  77.367  -1.956   0.018   0.088   0.893
E H  1VFBA                 30    0.317  57.662  -1.260   0.020   0.205   0.775
B N  1VFBA                 31    0.255  37.317  -0.798   0.022   0.359   0.619
B Y  1VFBA                 32    0.096  20.408   0.473   0.023   0.655   0.322
B L  1VFBA                 33    0.033   6.006   0.024   0.021   0.756   0.223
B A  1VFBA                 34    0.076   8.386  -0.089   0.018   0.846   0.136
B W  1VFBA                 35    0.035   8.442  -0.152   0.021   0.756   0.223
B Y  1VFBA                 36    0.045   9.681   0.326   0.021   0.756   0.223
B Q  1VFBA                 37    0.155  27.594   0.288   0.023   0.655   0.322
B Q  1VFBA                 38    0.092  16.503   0.180   0.022   0.359   0.619
E K  1VFBA                 39    0.399  82.033   1.410   0.005   0.045   0.951
E Q  1VFBA                 40    0.378  67.582  -0.034   0.005   0.015   0.979
E G  1VFBA                 41    0.784  61.709  -1.979   0.003   0.003   0.994
E K  1VFBA                 42    0.455  93.491  -0.183   0.018   0.019   0.964
E S  1VFBA                 43    0.369  43.282  -0.273   0.018   0.047   0.935
B P  1VFBA                 44    0.101  14.389   0.248   0.004   0.197   0.799
E Q  1VFBA                 45    0.279  49.865   0.536   0.003   0.718   0.279
B L  1VFBA                 46    0.087  15.893   0.487   0.001   0.900   0.099
B L  1VFBA                 47    0.029   5.273   0.636   0.001   0.900   0.099
B V  1VFBA                 48    0.019   2.936   0.707   0.001   0.959   0.040
B Y  1VFBA                 49    0.093  19.789  -0.056   0.001   0.959   0.040
B Y  1VFBA                 50    0.100  21.413  -0.332   0.001   0.900   0.099
B T  1VFBA                 51    0.158  21.970  -0.242   0.004   0.616   0.381
B T  1VFBA                 52    0.192  26.658  -1.063   0.005   0.262   0.733
E T  1VFBA                 53    0.559  77.575  -1.244   0.004   0.085   0.910
E L  1VFBA                 54    0.412  75.419  -0.070   0.018   0.047   0.935
E A  1VFBA                 55    0.571  62.946  -1.655   0.018   0.047   0.935
E D  1VFBA                 56    0.728 104.862  -1.157   0.018   0.047   0.935
E G  1VFBA                 57    0.593  46.693  -1.218   0.018   0.047   0.935
E V  1VFBA                 58    0.288  44.250   0.211   0.005   0.045   0.951
E P  1VFBA                 59    0.619  87.779  -0.194   0.005   0.045   0.951
E S  1VFBA                 60    0.394  46.153  -1.389   0.004   0.138   0.858
B R  1VFBA                 61    0.260  59.563  -0.180   0.004   0.616   0.381
B F  1VFBA                 62    0.054  10.758  -0.680   0.001   0.900   0.099
B S  1VFBA                 63    0.200  23.487  -0.198   0.001   0.900   0.099
B G  1VFBA                 64    0.123   9.696  -3.197   0.001   0.900   0.099
E S  1VFBA                 65    0.373  43.680   0.280   0.002   0.816   0.182
E G  1VFBA                 66    0.382  30.063  -0.461   0.004   0.514   0.481
E S  1VFBA                 67    0.609  71.351  -1.231   0.004   0.085   0.910
E G  1VFBA                 68    0.713  56.105  -0.880   0.005   0.015   0.979
E T  1VFBA                 69    0.426  59.072  -0.322   0.004   0.197   0.799
E Q  1VFBA                 70    0.378  67.564   0.637   0.002   0.816   0.182
B Y  1VFBA                 71    0.072  15.365  -0.275   0.001   0.900   0.099
B S  1VFBA                 72    0.244  28.562   1.013   0.006   0.962   0.032
B L  1VFBA                 73    0.030   5.511   0.458   0.001   0.959   0.040
E K  1VFBA                 74    0.293  60.291   1.046   0.001   0.959   0.040
B I  1VFBA                 75    0.072  13.283   0.201   0.002   0.816   0.182
E N  1VFBA                 76    0.459  67.168   0.546   0.004   0.514   0.481
E S  1VFBA                 77    0.533  62.444  -0.245   0.020   0.205   0.775
B L  1VFBA                 78    0.156  28.527   0.338   0.018   0.088   0.893
E Q  1VFBA                 79    0.460  82.102   0.673   0.018   0.088   0.893
E P  1VFBA                 80    0.446  63.245  -0.436   0.018   0.088   0.893
E E  1VFBA                 81    0.525  91.787  -0.740   0.053   0.043   0.903
B D  1VFBA                 82    0.209  30.189  -0.395   0.052   0.084   0.864
B F  1VFBA                 83    0.251  50.456   0.130   0.066   0.296   0.638
B G  1VFBA                 84    0.054   4.273  -0.233   0.021   0.756   0.223
B S  1VFBA                 85    0.185  21.623   0.806   0.018   0.846   0.136
B Y  1VFBA                 86    0.043   9.275   1.059   0.018   0.846   0.136
B Y  1VFBA                 87    0.068  14.467   0.906   0.021   0.756   0.223
B C  1VFBA                 88    0.020   2.780   1.208   0.023   0.655   0.322
B Q  1VFBA                 89    0.044   7.841  -0.272   0.023   0.655   0.322
B H  1VFBA                 90    0.109  19.900   0.047   0.022   0.552   0.426
B F  1VFBA                 91    0.248  49.693  -0.341   0.021   0.279   0.699
E W  1VFBA                 92    0.505 121.501  -1.558   0.018   0.088   0.893
E S  1VFBA                 93    0.482  56.514  -2.074   0.005   0.045   0.951
E T  1VFBA                 94    0.345  47.879  -1.002   0.018   0.088   0.893
E P  1VFBA                 95    0.415  58.945   0.029   0.020   0.205   0.775
B R  1VFBA                 96    0.237  54.296   0.365   0.021   0.451   0.528
B T  1VFBA                 97    0.137  19.002  -0.870   0.022   0.552   0.426
B F  1VFBA                 98    0.119  23.843  -0.678   0.004   0.514   0.481
B G  1VFBA                 99    0.119   9.373  -2.738   0.005   0.262   0.733
E G  1VFBA                100    0.458  36.029  -0.501   0.004   0.085   0.910
E G  1VFBA                101    0.328  25.837  -1.941   0.004   0.197   0.799
B T  1VFBA                102    0.156  21.609  -0.120   0.004   0.420   0.576
E K  1VFBA                103    0.395  81.354   1.612   0.001   0.900   0.099
B L  1VFBA                104    0.108  19.738  -0.012   0.001   0.959   0.040
E E  1VFBA                105    0.326  57.040   0.974   0.001   0.900   0.099
B I  1VFBA                106    0.131  24.253  -0.117   0.001   0.900   0.099
E K  1VFBA                107    0.698 143.496   1.402   0.004   0.197   0.799

Explain the output.  Go back.
```

---
